# The small-molecule protein ligand interface stabiliser E7820 induces differential cell line specific responses of integrin α2 expression

**DOI:** 10.1186/s12885-021-08301-w

**Published:** 2021-05-18

**Authors:** Michael David Hülskamp, Daniel Kronenberg, Richard Stange

**Affiliations:** Department of Regenerative Musculoskeletal Medicine, Institute for Musculoskeletal Medicine, Westfälische Wilhelms-Universität Münster, Albert-Schweitzer Campus 1 Building W1, 48149 Münster, Germany

**Keywords:** E7820, Integrin α2, CAPERα, SPLINTS

## Abstract

**Background:**

The mechanism of small-molecule stabilised protein-protein interactions is of growing interest in the pharmacological discovery process. A plethora of different substances including the aromatic sulphonamide E7820 have been identified to act by such a mechanism. The process of E7820 induced CAPERα degradation and the resultant transcriptional down regulation of integrin α2 expression has previously been described for a variety of different cell lines and been made responsible for E7820’s antiangiogenic activity. Currently the application of E7820 in the treatment of various malignancies including pancreas carcinoma and breast cancer is being investigated in pre-clinical and clinical trials. It has been shown, that integrin α2 deficiency has beneficial effects on bone homeostasis in mice. To transfer E7820 treatment to bone-related pathologies, as non-healing fractures, osteoporosis and bone cancer might therefore be beneficial. However, at present no data is available on the effect of E7820 on osseous cells or skeletal malignancies.

**Methods:**

Pre-osteoblastic (MC3T3 and Saos-2) cells and endothelial (eEnd2 cells and HUVECs) cells, each of human and murine origin respectively, were investigated. Vitality assay with different concentrations of E7820 were performed. All consecutive experiments were done at a final concentration of 50 ng/ml E7820. The expression and production of integrin α2 and CAPERα were investigated by quantitative real-time PCR and western blotting. Expression of CAPERα splice forms was differentiated by semi-quantitiative reverse transcriptase PCR.

**Results:**

Here we present the first data showing that E7820 can increase integrin α2 expression in the pre-osteoblast MC3T3 cell line whilst also reproducing canonical E7820 activity in HUVECs. We show that the aberrant activity of E7820 in MC3T3 cells is likely due to differential activity of CAPERα at the integrin α2 promoter, rather than due to differential CAPERα degradation or differential expression of CAPERα spliceforms.

**Conclusion:**

The results presented here indicate that E7820 may not be suitable to treat certain malignancies of musculoskeletal origin, due to the increase in integrin α2 expression it may induce. Further investigation of the differential functioning of CAPERα and the integrin α2 promoter in cells of various origin would however be necessary to more clearly differentiate between cell lines that will positively respond to E7820 from those that will not.

**Supplementary Information:**

The online version contains supplementary material available at 10.1186/s12885-021-08301-w.

## Introduction

In recent years small-molecule protein ligand interface stabilisers (SPLINTS) have become a topic of increased interest and research, since various previously approved and marketed drugs, such as lenalidomide, which has shown strong activity against haematological malignancies, were shown to act by this mechanism [1, 2]. SPLINTS act by stabilising protein-protein interactions (PPIs) and have been identified in a large variety of different signalling pathways [3].

The aromatic sulphonamide E7820 (N-(3-Cyano-4-methyl-1H-indol-7-yl)-3-cyanobenzene-sulfonamide) was initially characterised as a novel modulator of integrin α2 expression and shown to decrease integrin α2 mRNA levels and integrin α2 expression on HUVEC cell lines [4]. Since its first publication E7820 was shown to possess anti-cancer activity in vitro and in vivo and has been tested in phase I and phase II clinical trials [5–9]. More recently it was shown to act by stabilising PPIs and thus identified as belonging to the SPLINTS [3, 10, 11].

**Fig. 1:**
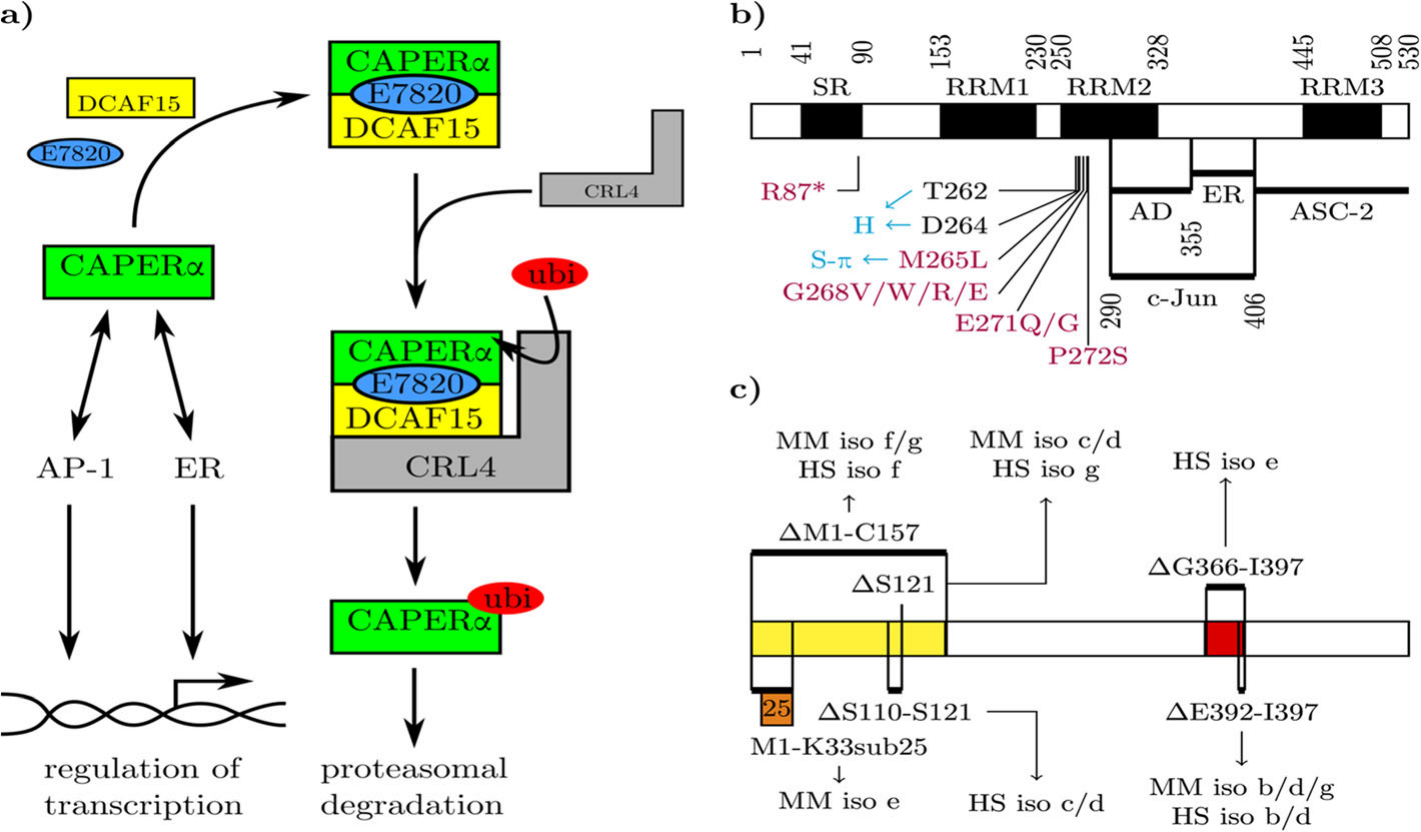
Illustration of CAPERα interactions and structure. **a** Interactions of CAPERα with DCAF15 mediated by E7820 via a molecular glue mechanism leads to polyubiquitinylation (ubi) of the former by cullin-RING ubiquitin ligase 4 (CRL4) and subsequent proteasomal degradation [12, 13]. This impedes the interaction between CAPERα and activator protein-1 (AP-1) as well as estrogen receptor (ER) [14], both of which represent plausible links to the regulation of integrin α2 expression [15, 16]. **b** Domain structure of CAPERα adapted from Jung et al. showing a serine/arginine-rich domain (SR) and three RNA-recognition motifs (RRM1–3) [14, 17]. The interaction sites with the c-Jun domain of activator protein 1 (AP-1), with oestrogen receptor α and β (ER) and with activating signal co-integrator 2 (ASC-2) are indicated [14]. Purple labels indicate mutations found in E7820 resistant cell lines [12, 17]. Blue labels show the molecular interactions of specific amino acids with E7820 [10]. **c** Structure of different CAPERα isoforms. The variable n-terminal domain (yellow) may be entirely deleted (ΔM1-C157), contain a substitution of an alternate 25 amino acids for the initial 33 n-terminal amino acids (M1-K33sub25), lack 12 amino acids from serine 110 to serine 121 (ΔS110-S121), or lack serine 121 only (ΔS121). The MII-2 domain (red) may be completely (ΔG366-I397) or partly (ΔE392-I397) missing. The murine (MM) and human (HS) isoforms (iso) that contain each of these features are indicated

E7820 acts as a “molecular glue” by stabilising the formation of a complex between co-activator of activating protein 1 and oestrogen receptors (CAPERα) and DDB-1 and cullin-4 associated factor 15 (DCAF15), resulting in the increased proteasomal degradation of the former [12]. In this process, DCAF15 serves as an adaptor conferring substrate specificity to cullin-RING ubiquitin ligase 4 (CRL4), as is illustrated in Fig. 1a [13]. CRL4 has been shown to be widely expressed and play an important role in many physiological cellular processes as well as in tumourigenesis [18]. Canonical E7820 activity (reduction of integrin α2 expression) has been shown in human endothelial [4], haematopoietic and gastrointestinal epithelial cells [12] as well as murine haematopoietic cells [5]; although cell type specificity of the action of E7820 has previously been proposed, no data to this effect has been published and the basis for it has not been elucidated thus far [4].

The genomic, RNA and protein sequences of CAPERα can be found in the NCBI Gene database [19] for *Mus musculus* (MM) under Gene ID: 170791 and for *Homo sapiens* (HS) under Gene ID: 9584. Supplementary Table 3 gives an overview of the different mRNA and protein isoforms. Figure 1b shows the functional domains of CAPERα and correlates these with the features of the different isoforms in part c. The locations of mutations found in cell lines resistant to E7820 induced cell death (purple in Fig. 1 [12, 17]), closely coincides with the position of the amino acid residues known to interact with E7820 (blue [10]), which suggests that resistance to degradation may be the key mechanism governing E7820 resistance. This is supported by the previously published dose response analyses of mutant as well as DCAF15^−/−^ cells compared to parental cell lines [12].

Whilst the molecular mechanism of CAPERα degradation in response to E7820 has been elucidated in great detail [10, 12], the same cannot be said for the effects downstream of CAPERα. CAPERα is known to interact directly with oestrogen receptor α and β (ER) and with the c-Jun component of activating protein 1 (AP-1) via interaction domains found between amino acids 356 and 400 and amino acids 290 and 400 of the full length protein respectively and enhances their transactivational activities [14].

More recently, short peptide fragments corresponding to the amino acids 356 to 400 of full length HCC1.3 and HCC1.4 (*Homo sapiens* CAPERα isoforms a and b respectively) were used to modulate CAPERα and AP-1 activity in triple negative breast cancer cells. This modulation was different between the two peptides and also between different cell lines even within the same tumour entity [9]. This is plausible since the functionality of AP-1 has been shown to be dependent on the exact composition of the Jun-Fos dimer [20], which may well be different in different cell lines. Together these reports indicate that CAPERα may act independently and differentially via ER and via AP-1. Both AP-1 and ERα have been implicated in the regulation of integrin α2 expression which has also shown to be cell type and phase specific [15, 16, 21].

Despite the unclear downstream mechanism of action of CAPERα various groups have been able to show decreased integrin α2 expression in response to E7820 via CAPERα degradation and linked it to, among others, reduced pulmonary metastasis of xenograft HCT-116 cells [22] and to reduce tumour volume as well as reduced tumour associated angiogenesis in non-small cell lung cancer xenograft models [23], the latter of which is likely conferred at least in part by reduced integrin α2 levels [24].

Integrin α2 expression on osteoblasts has also been identified by our group as a promising target to modulate bone homoeostasis and potentially treat conditions due to age related bone deterioration [25]. The integrin α2 knockout mouse was shown to have an increased amount of trabecular bone and improved biomechanical properties, which was linked to increast osteoblast activity [25].

Together the previously published data identifies E7820 induced changes in integrin α2 expression via CAPERα degradation as a promising target for investigation in bone. Here we therefore analysed the effect of E7820 on integrin α2 expression in human and murine osseous and endothelial cell lines.

## Materials and methods

### Cell culture

MC3T3 cells [26] were available in the lab having previously been kindly provided by Prof. Hartmann (Department of Bone and Skeletal Research, Institute for Musculoskeletal Medicine, University Hospital Münster, Westfälische Wilhelms-Universität Münster, 48,149 Münster, Germany). They were cultured in α-MEM (PAN Biotech) containing 10% FBS (Gibco, Lot: 42F2567K) and 2 mM L-glutamine and were used in passages 35 to 45. Their pre-osteoblast identity was subsequently confirmed by qRT-PCR and calcification assays under differentiation induced by β-glycerolphosphate, ascorbic acid and dexamethasone as described previously [25]. Soas-2 cells [27] (Sigma, 89,050,205, used in passage 20 to 30) and eEnd2 cells [28] (kindly provided by Prof. Hallmann, Institute of Physiological Chemistry and Pathobiochemistry, Westfälische Wilhelms-Universität Münster, 48,149 Münster, Germany) were cultured in DMEM (Sigma) containing 10% FBS and used within 10 passages. Human umbilical vein endothelial cells (HUVECs) were kindly provided by Prof. Schnittler (Institute of Anatomy and Vascular Biology, Westfälische Wilhelms-Universität Münster, 48,149 Münster, Germany) having been isolated from three independent donor cords as previously described [29] in accordance with the approval given by the ethics board of the University of Münster (2009–537-f-S) and confirmed HIV and hepatitis B free. They were cultured on rat tail collagen type I (Corning, Lot: 6116001) coated culture plates in M-199 (Sigma) supplemented with 10% heat inactivated FBS, 2 mM L-glutamine, 15 mM HEPES and 50 μg/ml ECGS (Sigma, Lot: SLBV3810) and used in the second passage. 24 h prior to and during stimulation HUVECs were cultured in the standardised Endothelial Cell Growth Medium (PromoCell) without antibiotics. If not stated otherwise, all media were supplemented with 10 U/ml penicillin 10 μg/ml streptomycin (Merck) and cells were incubated at 37 °C and 5% CO2 in humidified atmosphere. Tests for mycoplasm contamination were performed using the Venor®GeM Classic kit and the MD Taq Polymerase (Minerva Biolabs) according to the manufacturer’s instrucitons.

N-(3-Cyano-4-methyl-1H-indol-7-yl) -3-cyano-benzene-sulfonamide (E7820) was purchased from Hölzel-Biotech and phorbol 12-myristate 13-acetate (PMA) was purchased from LC Laboratories®; both were dissolved in DMSO (Sigma). DMSO concentration in culture media was controlled throughout and never exceeded 0.3%.

In all assays analysing the expresion of integrin α2 expression PMA was used as a positive control. It is known to increase integrin α2 expression [21], likely via the protein kinase C pathway [30] and has been used in previous research into the activity of E7820, where it was used to recover decreased integrin α2 expression after treatment of HUVECs with E7820 [4] and into the functioning of the integrin α2 promotor [21]. In order to most closely reproduce this data, combined treatment with E7820 and PMA was used as control for endothelial cells. PMA alone was used as a control for osteoblastic cells, since preliminary experiments had shown E7820 to increase integrin α2 expression in these cells, thus making a recovery, such as was observed for endothelial cells, impossible.

### Cytotoxicity assay

Cytotoxicity was assessed using the well-established MTT assay [31] as a conjugate measure for cell proliferation, viability and metabolic activity. Briefly, 10% (v/v) of MTT Reagent (1 mg/ml MTT in PBS) was added the cells incubated at 37 °C for 2 h. The reaction product was dissolved in 10% (m/v) SDS with 50% (v/v) dimethyl formamide in distilled water. Quantification was performed by absorption measurement at 550 nm with 630 nm reference wavelength using the Spark 10 M microplate reader (Tecan). The cells were also monitored for morphological changes using the CKX41 inverted phase contrast microscope (Olympus).

### Quantitative reverse transcription polymerase chain reaction (qRT-PCR)

RNA for qRT-PCR was prepared using the RNeasy Mini or Micro Kits (QIAGEN) as dictated by expected RNA yield. Cells were lysed in RT-buffer supplemented with 1% (v/v) β-mercaptoethanol. The purification including a DNAse digest using the RNAse free DNAse Set (QIAGEN) was performed according to the manufacturers’ instructions. RNA concentration was measured by UV spectroscopy using the Eppendorf Bio Photometer and μCuvette G1.0. 500 ng total RNA was used to produce copy DNA (cDNA) for each sample using the High Capacity RNA-to-cDNA-Kit (Thermo Fischer Scientific) according to the manufacturer’s instructions. qRT-PCR was performed in the CFX384 Real-Time System (Bio-Rad) with the primers listed in supplementary Table S1 using the SYBR Fast Universal Kit (Kapa Biosystems) according to the manufacturer’s instructions. All primers were tested for specificity by analysis of the melting curves and electrophoresis and for efficiency by measurement of serial cDNA dilutions. qRT-PCR data was quantified by the ΔΔCt method relative to GAPDH and HPRT as housekeeping controls [32].

### Semi-quantitative reverse transcription polymerase chain reaction (sqRT-PCR)

Preparation of RNA and cDNA was performed as described for qRT-PCR. sqRT-PCR was performed using the peqGOLD Taq all-inclusive Kit (Peqlab) and the primers listed in supplementary Table S2 in the peqSTAR 2X thermal cycler (VWR international) according to the manufacturer’s instructions. Electrophoresis was performed in a 2% agarose gel (peqGOLD universal-agarose, VWR chemicals) containing 1x GelRed® nucleic acid gel stain (Biotium) and the bands were visualised using the FAS-Digi Pro gel documentation system (NIPPON genetics). Band intensities were analysed using the gel analysis functionality of ImageJ (open source image processing software, version 2.0.0-rc-69/1.52i).

### Quantitative fluorescent linked western blot

Samples were prepared for western blot analysis by lysis in RIPA buffer (150 mM NaCl, 1% (v/v) Triton × 100, 0.5% (m/v) sodium deoxycholate, 0.1% (v/v) SDS, 50 mM Tris Base, pH 8). Samples containing 70 μg total protein each were precipitated using trichloroacetic acid and re-dissolved in 15 μl SDS-sample buffer (252 mM Tris, 40% Glycerol (v/v), 8% SDS (m/v), 0.04% (m/v) bromophenol blue, pH 6.8). If necessary, the sample pH was adjusted through addition of single Tris-Base crystals until the indicator was a bright blue colour. The sample was then denatured for 5 min at 95 °C.

4.5% stacking and 10% running gels were used. The bands were transferred to Immobilon®-FL transfer membrane (Merck) using semi-dry blotting in the trans-blot® Turbo transfer system (bio-rad) with a discontinuous transfer buffer system (Tris-CAPS-methanol buffer at the anode and Tris-CAPS-SDS at the cathode: 60 mM Tris, 40 mM CAPS, pH 9.6, plus either 15% (v/v) methanol or 0.1% (m/v) SDS respectively) at 25 V limited to 1 a for 30 min.

The resulting blots were blocked using 10% (m/v) BSA in TBS and stained with sheep-anti-integrin α2 (R&D Systems, AF1740, 1:500), mouse-anti-CAPERα (Santa Cruz Biotechnology, sc-376,531, 1:2000) and rabbit-anti-GAPDH (Proteintech, 10,494–1-AP, 1:2000) antibodies in 5% (m/v) BSA in TBS containing 0.2% (v/v) Tween®-20. DyLight-800 labelled donkey-anti-goat (Li-Cor, 925–32,214, 1:20,000) and goat-anti-mouse (Li-Cor, 925–32,210, 1:20,000) as well as Dylight-680 labelled goat-anti-rabbit (Li-Cor, 925–68,071, 1:20,000) were used as secondary antibodies in 1% (m/v) BSA in TBS containing 0.2% (v/v) Tween-20 and 0.01% (m/v) SDS. The blots were imaged on the Odyssey® CLx imaging system (Li-Cor) and analysed using the associated Image Studio™ software (Li-Cor, Version 5.2.5).

### Statistical analysis

Statistical analysis and graphical illustration were performed using GraphPad Prism software (Graph Pad Software Inc., Version 8.2.0). Data from the MTT assay was analysed using sigmoidal 4PL or linear regression as appropriate. Comparisons between > 2 groups were performed using ANOVA followed by multiple comparison. All other data was analysed by one-sample and two-sample two-tailed t-tests as appropriate. Significance levels were defined as * *p* < 0.05, ** *p* < 0.01, *** *p* ≤ 0.001.

## Results

### Cytotoxicity assay

**Fig. 2:**
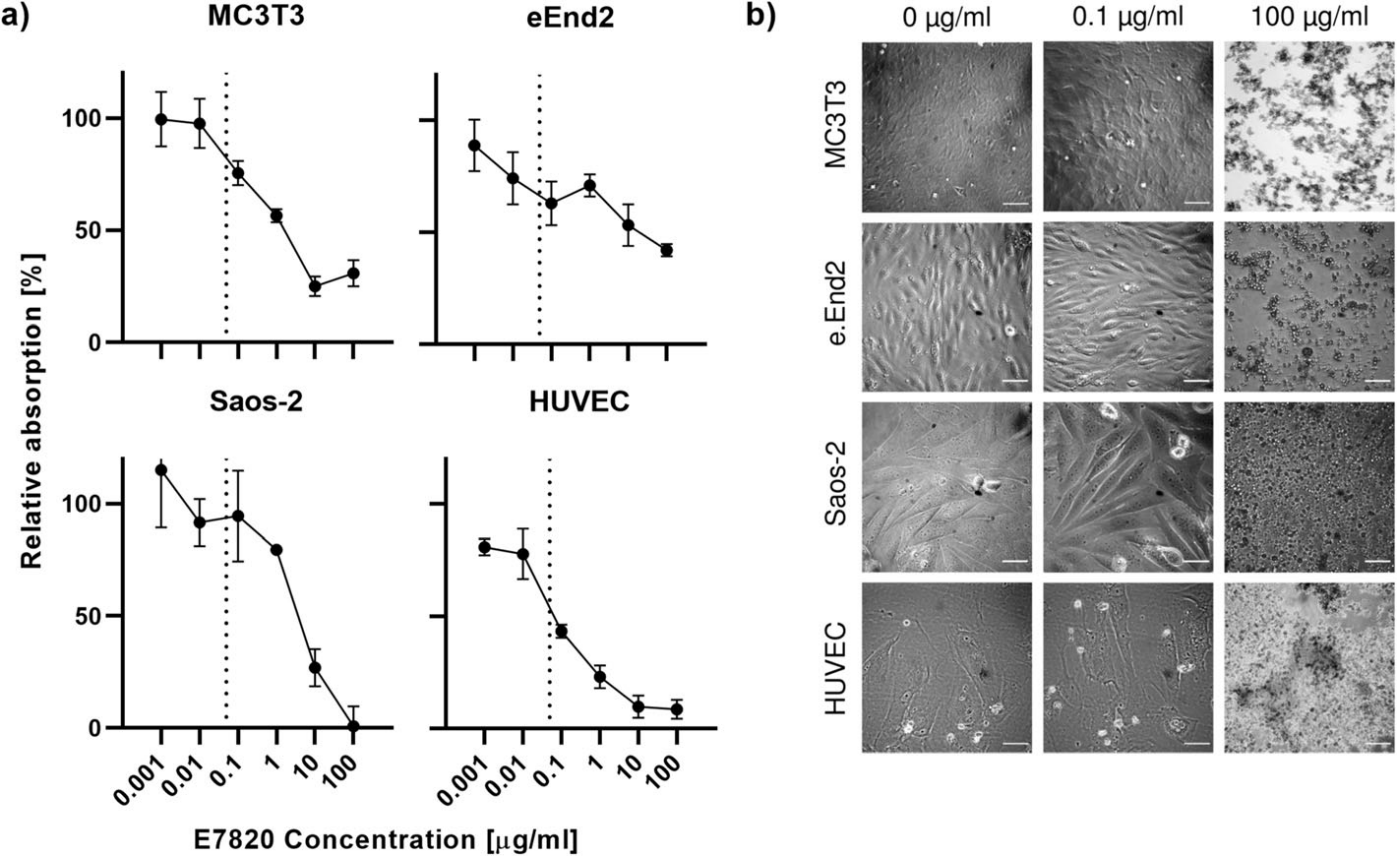
Analysis of cytotoxicity of E7820 on MC3T3, Saos-2, eEnd2 cells and HUVECs at concentrations ranging from 0.001–100 μg/ml after 3 days of incubation; **a** MTT assay quantified as percentage absorption relative to solvent control (DMSO), dotted line indicates 50 ng/ml; **b** phase contrast microscopic images of the cells under control conditions, 0.1 μg/ml and 100 μg/ml E7820. Scale bar = 40 μm

In order to establish a safe working concentration for E7820 in cell culture an MTT assay was performed for all cell lines at concentrations ranging from 0.001–100 μg/ml. Absorption decreased at higher E7820 concentrations in all cases, as can be seen in Fig. 2a. The profile of the curves was sigmoidal in nature for MC3T3, Saos-2 cells and HUVECs and more linear for eEnd2 cells. 4-PL and linear regression showed the half-maximal absorption to be reached at 0.4 μg/ml, 66.6 μg/ml, 4.2 μg/ml and 0.1 μg/ml E7820 for MC3T3, eEnd2, Saos-2 cells and HUVECs respectively.

Figure 2b shows phase contrast microscopic images of all cell lines under control conditions (0 μg/ml E7820) as well as 0.1 μg/ml and 100 μg/ml E7820. No morphological difference was visible between cells under the two foremost of these conditions, while only cell debris was visible at 100 μg/ml E7820 for all cell lines tested here.

### Effect of E7820 on integrin expression

**Fig. 3:**
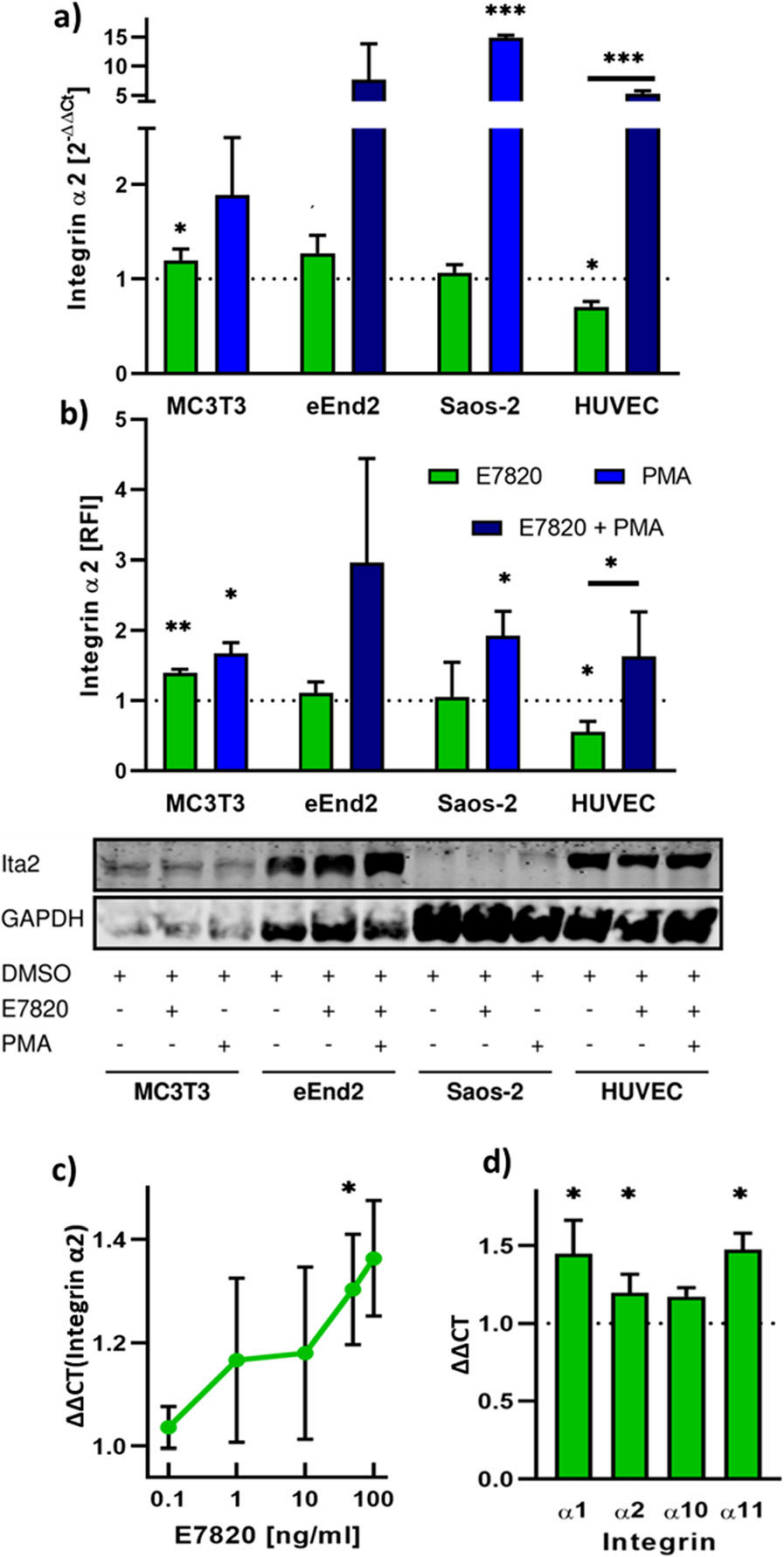
Effect of E7820 on integrin α2 expression. **a** Relative integrin α2 mRNA expression as determined by qRT-PCR after 17 h incubation with 50 ng/ml E7820 and 100 nM PMA or 50 ng/ml E7820 + PMA 100 nM PMA respectively, quantified using GAPDH and HPRT as housekeeping controls relative to solvent control (DMSO). **b** Relative integrin α2 protein expression as determined by quantitative fluorescent linked western blot analysis after 3 days of incubation with DMSO control, 50 ng/ml E7820, 100 nM PMA and 50 ng/ml E7820 + PMA 100 nM PMA and exemplary integrin α2 western blot. The full blot is shown in Figure S1. **c** Dose response relationship between E7820 concentration and integrin α2 mRNA expression in MC3T3 cells quantified as described above. **d** Change in expression of the collagen binding integrins α1, α2, α10 and α11 in response to 50 ng/ml E7802. All data is the mean of ≥3 independent repeats +1SD. * *p* < 0.05, ** *p* < 0.01, *** *p* < 0.001

Integrin α2 mRNA expression in MC3T3, eEnd2, Saos-2 cells and HUVECs incubated with 50 ng/ml E7820 for 17 h was analysed by qRT-PCR relative to solvent control (Fig. 3a). eEnd2 and Saos-2 cells did not show a significant change in expression. HUVECs showed a significant reduction of integrin α2 mRNA to approximately 70% (*p* = 0.011) of control whilst MC3T3 cells showed an increase in integrin α2 mRNA of approximately 20% (*p* = 0.047). Incubation with 100 nM PMA led to an increase in integrin α2 expression in all cell lines and was able to override the reduced expression observed under treatment with 50 ng/ml E7820 in HUVECs. This PMA-induced increase in integrin α2 expression was statistically significant for Saos-2 cells and HUVECs (*p* ≤ 0.001 in both cases) but not quite significant for MC3T3 (*p* = 0.062) or eEnd2 (*p* = 0.081) cells.

The observed effect of E7820 on integrin α2 expression in MC3T3 cells was validated by dose response analysis at concentrations ranging from 0.1–100 ng/ml. A clear dose response relationship was obtained (Fig. 3c); the change in expression was statistically significant at 50 ng/ml (*p* = 0.039) and 100 ng/ml (*p* = 0.030).

In order to confirm the effect observed on the mRNA level, quantitative western blot analysis of integrin α2 against GAPDH as housekeeping control was performed after 3 days of incubation under the same conditions used above. The results as well as sections of a representative blot are shown in Fig. 3b. Increased levels of integrin α2 in response to stimulation with PMA were found in all cell lines. This increase was significant for MC3T3, Saos-2 cells and HUVECs (*p* = 0.017, *p* = 0.046 and p = 0.046 respectively). It was not quite significant for e.End2 cells (*p* = 0.097). Furthermore, the decreased expression in HUVECs and the increased expression in MC3T3 cells under stimulation with E7820 were confirmed (56 and 140% of control at *p* = 0.037 and *p* = 0.006 respectively).

The expression of the collagen binding integrins (integrin α1β1, α2β1, α10β1 and α11β1) in MC3T3 cells under stimulation with 50 ng/ml E7820 after 17 h was also analysed by qRT-PCR. A consistent tendency towards upregulation of these integrins was observed and found to be significant for integrin α2, α10 and α11 (*p* = 0.047, *p* = 0.038 and *p* = 0.017 respectively) and not quite significant for integrin α1 (*p* = 0.067), which may, however, be due to increased variance in the data for this measurement (Fig. 3d).

### Analysis of E7820 interaction partners

**Fig. 4:**
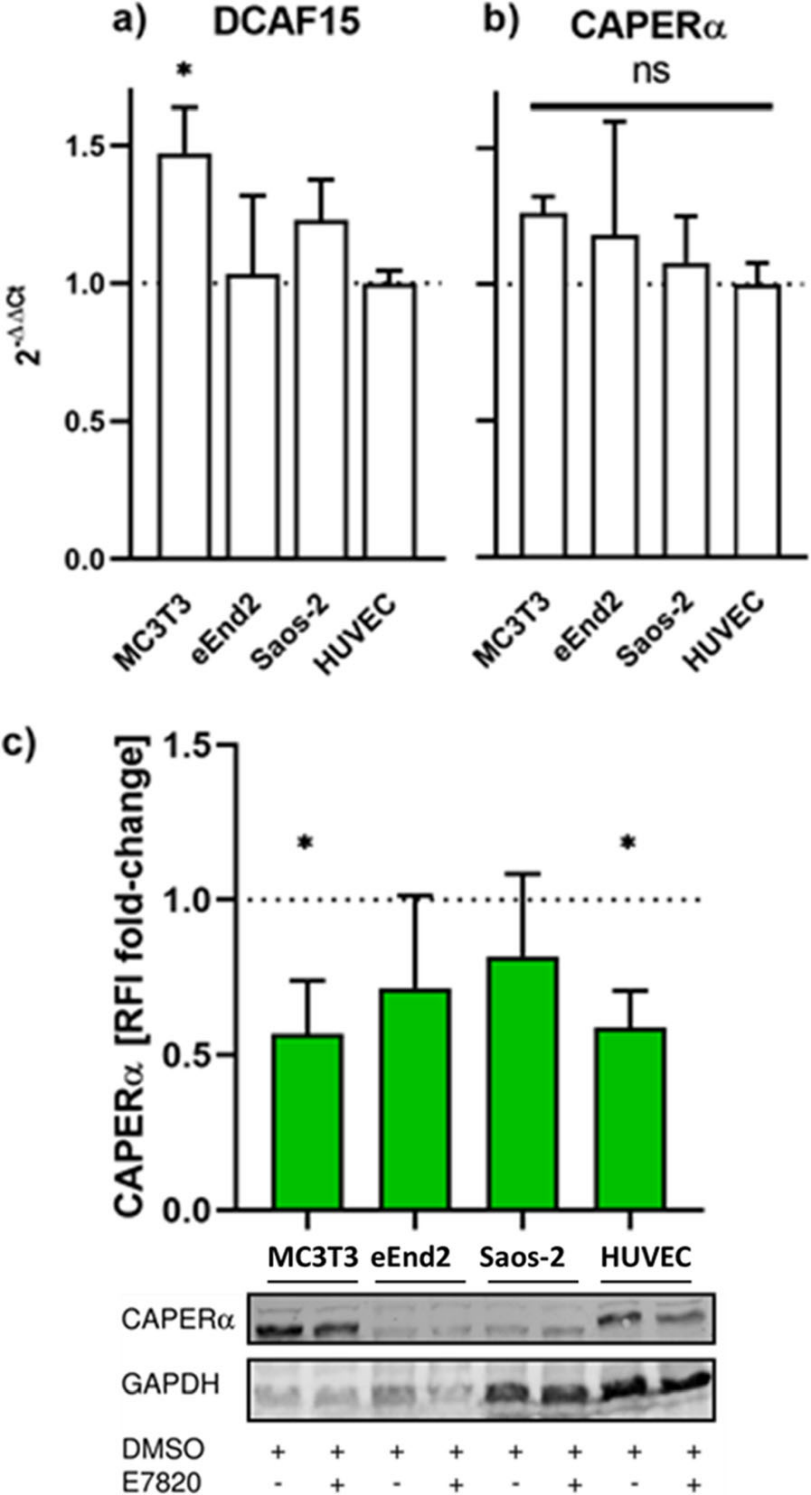
E7820 dependent degradation of CAPERα; Relative basal expression of **a** DCAF15 and **b** CAPERα as determined by qRT-PCR quantified using GAPDH and HPRT as housekeeping controls relative to basal expression in HUVECs. **c** Quantification of fluorescent linked western blots of CAPERα under treatment with 50 ng/ml E7820 for 3 days relative to respective solvent controls and a representative western blot. The full blot is shown in Figure S2. GAPDH is used as loading control. All data is the mean of ≥3 independent repeats +1SD. * *p* < 0.05, ** *p* < 0.01, *** *p* < 0.001

In order to assess for possible differences in expression levels of the two binding partners of E7820, DCAF15 and CAPERα, which could potentially explain the abarant activity of E7820 in MC3T3 cells, the basal expression of these was analysed by qRT-PCR for the different cell lines (Fig. 4a and b). A one-way ANOVA for DCAF15 expression was significant (*p* = 0.044) and subsequent multiple testing showed that the expression in MC3T3 cells was significantly higher (*p* = 0.013) than in the other cell lines. The ANOVA for CAPERα was not significant (*p* = 0.531). In order to confirm CAPERα degradation in all the cell lines a quantitative western blot analysis was performed after 3 days of incubation of all cell lines with 50 ng/ml E7820 and respective solvent controls (Fig. 4c). CAPERα levels after treatment with E7820 were consistently reduced for MC3T3, eEnd2 and Saos-2 cells as well as HUVECs (57, 72, 82 and 59% of control respectively). This decrease was statistically significant for MC3T3 cells and HUVECs (*p* = 0.049 and *p* = 0.027 respectively) and not quite significant for eEnd2 and Saos-2 cells.

### Analysis of CAPERα isoform expression

**Fig. 5:**
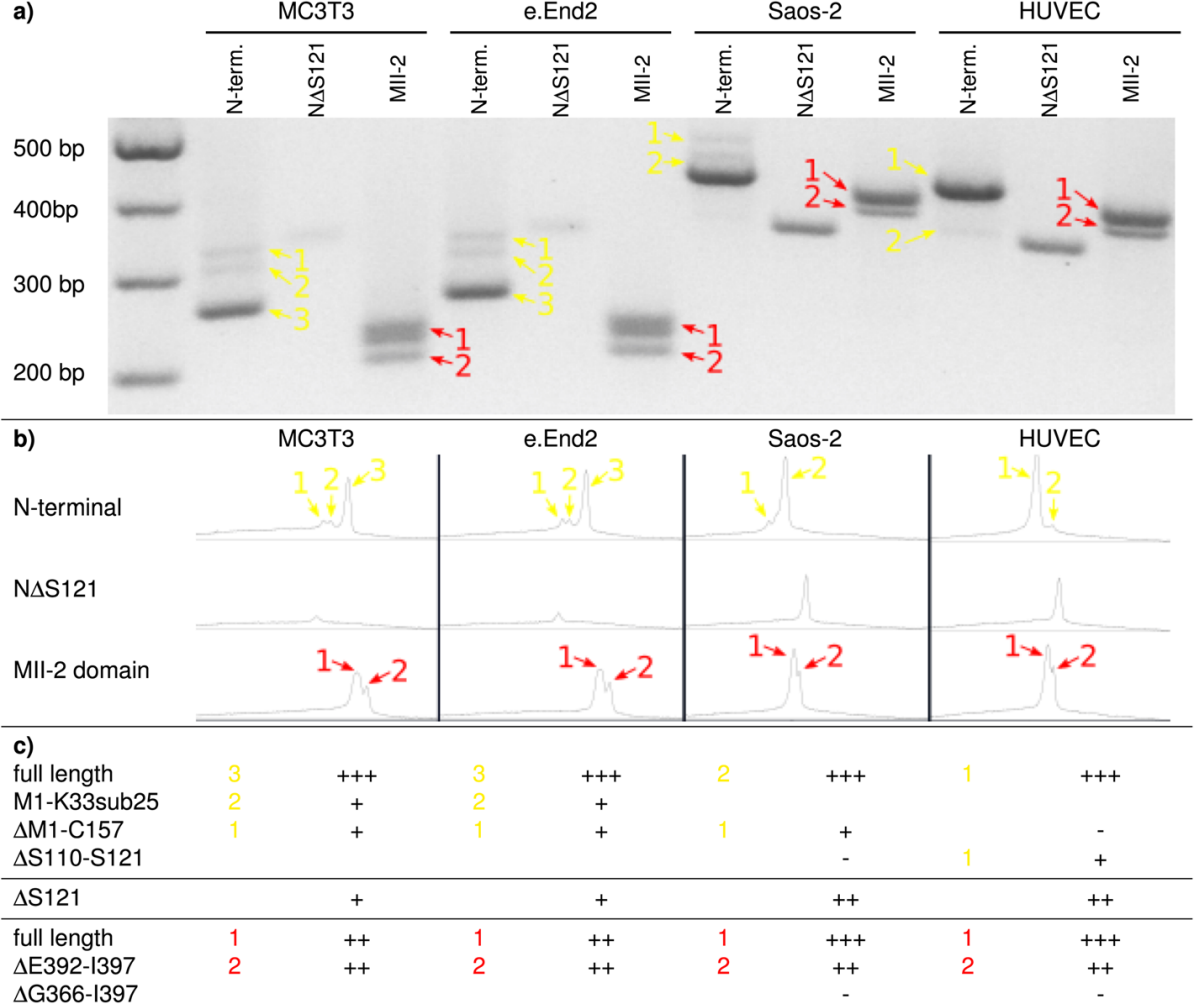
Semi-quantitative reverse transcription PCR (sqRT-PCR) of CAPERα isoform expression in MC3T3, eEnd2, Saos-2 cells and HUVECs under standard culture conditions. **a** Image of a representative agarose gel of three independent repeats. **b** Intensity profiles of the lanes of the agarose-gel as obtained using the ImageJ gel analysis functionality. **c** Quantification of band intensities as strongly positive (+++, ≥ 2/3 of maximal intensity), positive (++, < 2/3 and ≥ 1/3 of maximal intensity), weakly positive (+, < 1/3 of maximal intensity) and negative (−, no band detectable). Yellow annotations were used for the n-terminal primer pairs and red annotations were used for the MII-2 domain primer pairs

Since no major difference in the expression of E7820s interaction partners and in CAPERα degradation could be detected between the cell lines, the basal expression of different CAPERα isoforms, the presence of which presents a further potential explaination for the observed aberant activity of E7820, was analysed by sqRT-PCR. Figure 5a shows one agarose gel of three independent repeats using the primers indicated, Fig. 5b shows the intensity profiles of each lane and 5c shows the resulting ordinal quantification of the expression of the various splice variants. Yellow annotations were used for n-terminal domain variants and red annotations were used for MII-2 domain variants.

The strongest bands for the n-terminal primers in MM and HS were detected at 256 bp (yellow arrow 3 in MC3T3 and eEnd2 cells) and 420/423 bp (yellow arrow 2 in Saos-2 cells and HUVECs) respectively, which both correspond to the full length n-terminal domain as found in MM and HS isoforms a and b as well as HS isoform e or the full length n-terminal domain with the deletion of serine 121 (ΔS121) as found in MM isoform d and the analogous MM isoform c and HS isoform g. Further weak bands, corresponding to the shortened or truncated n-terminal domain M1-K33sub25 (384 bp, yellow arrow 1 for MC3T3 and eEnd2), found exclusively in MM isoform e, and ΔM1-C157 (yellow arrow 2 at 329 bp in MC3T3, eEnd2 and at 496 bp in Saos-2 but not in HUVECs), found in MM and HS isoform f as well as in MM isoform g, were detected at approximately equal intensities. Using the primers for the variable MII-2 region, strong or moderately strong bands were detected at 214 bp and 196 bp for MC3T3 and eEnd2 cells and 398 bp and 380 bp for Saos-2 cells and HUVECs, corresponding to the full length and the ΔE392-I397 variants respectively in all of the cell lines. The 1 amino acid variant ΔS121 was detected at equal levels between the MM and between the HS cell lines at low and moderate levels respectively.

## Discussion

As results present themselves, E7820, which is a powerful effector of integerin α2 inhibiton in HUVECs, showed a different outcome in other cell types (murine and non-endothel cells).

Data from the cytotoxicity assay effectively showed that an E7820 concentration of 50 ng/ml was tolerated by all cell-lines used here and was therefore used for further analysis. The decrease in absorption at this concentration (indicated by dashed lines in 2a), which was most pronounced in the endothelial cell lines, is most likely due to reduced metabolic activity rather than due to cytotoxic effects of E7820, since homogenous growth patterns and no signs for apototic or necrotic cell death were observed at this concentration (Fig. 2b). The results closely recapitulate the findings of Uehara et al. for cell lines sensitive to CAPERα degradation by E7820, thus indicating that the cell lines used here are suitable for the investigation of transcriptional modulation by E7820.

Both the qRT-PCR and quantitative western blot data for integrin α2 expression in HUVECs showed the expected decrease in response to E7820, which has previously been described [4]. Supprisingly, integrin α2 expression in MC3T3 cells in response to E7820 was found to be significantly increased. This represents the first published instance of increased integrin α2 expression in response to E7820. Overall the qRT-PCR and western blot data is complementary, although the magnitude of the change in expression varied, which may be due to the different time points analysed or to cell line specific differences in mRNA processing and transcription.

Since the increase in integrin α2 expression in response to E7820 in MC3T3 cells was unexpected, efforts were undertaken to further confirm the veracity of this observation. Firstly, the clear dose response relationship between E7820 concentration and integrin α2 expression supports the idea of a specific interaction that is stable at varying concentrations, especially since such dose response relationships have previously been shown for various effects of E7820 [4, 12] as well as for other SPLINTS, such as lenalidomide [3, 33].

Secondly, the consistant upregulation of the collagen binding integrins (α1β1, α2β1, α10β1 and α11β1), partly redundant and compensatory functionality of which has previously been proposed [34], indicates that the increase in integrin α2 expression observed here was a direct effect of E7820; any secondary or compensatory change in integrin α2 expression would require the decreased expression of one of the other collagen binding integrins, to lead to a compensatory up-regulation of integrin α2 expression. This is in agreement with previous data published by our group, which showed that the expression of integrin α1, α10 and α11 was unchanged in both integrin α2 knockout osteoblasts and tenocytes [25, 35].

Furthermore, despite their cell type and differentiation specific expression [34], various structural and functional similarities have been identified between the promoters of these four integrin α-subunits. AP-1 and Sp1 consensus binding sites are present in the promoters of integrin α1 [36, 37], α2 [16] and α11 [38, 39] and Sp1 has been demonstrated to play a major role integrin α2 and α11 expression [39, 40]. A key driver of α10 integrin expression, retinoblastoma protein, has also been shown to interact with Sp1, although no direct activity of Sp1 on the α10 integrin has been demonstrated [41]. Such commonalities and mechanistic links could explain the general increased expression observed here as well as the likewise homodirectional modulation of the expression of other integrin α-subunits observed previously [4].

PMA provided an effective positive control and resulted in the appropriate increase in integrin α2 in all the cell lines and was able to override the reduced expression observed under treatment with 50 ng/ml E7820 in HUVECs. The large standard deviation observed for MC3T3 and eEnd2 cells appears to be due to larger natural variance in response to PMA in these cell lines, since the mean standard deviation of technical replicates was < 0.12 and thus below the variance observed in the data.

Together this data shows that E7820 increases integrin α2 expression in MC3T3 cells in a direct dose dependent manner, which represents a thus far undescribed aberant activity of E7820 in this cell line. To investigate the genesis of this effect, it was necessary to analyse the individual components involved in the process.

A first explaination of the observed data could be a differential effects of E7820 on CAPERα degradation in the different cell lines. No relevant difference between DCAF15 and CAPERα expression was found in the cell lines tested here and CAPERα was found to be consistantly reduced in response to E7820 simulation for both MC3T3 cells and HUVECs. Whilst the intensity of the GAPDH bands differed between the cell lines, it was consistant within each of these, thus allowing for quantification of expressional changes due to different incubation conditions within each cell line. The data therefore indicates that the process of E7820 stabilised complex formation between DCAF15 and CAPERα leading to proteasomal degradation of the latter occurs in MC3T3 cells and HUVECs. It remains likely that this process also occurs in eEnd2 and Soas-2 cells, although the effect could not be resolved to a statistically significant degree here. Structural data on E7820s interaction with CAPERα and the structure of its different isoforms (see Fig. 1) further supports this conclusion, since neither the mutation sites nor the amino acid residues known to interact with E7820 are part of the variable domains (with the exception of the R87* mutation, which through the introduction of a stop codon leads to a drastically shortened protein [12]). It therefore seems unlikely that the isoforms differ in regard to their susceptibility to E7820 mediated degradation. The variant activity of E7820 must therefore be explained by a process downstream of CAPERα degradation.

One mechanism that could potentially explain this differential activity may be the presence of different CAPERα isoforms in the different cell lines. It is possible that different CAPERα isoforms may act in different and even opposing manners. Such opposing functionality of different splice forms of the same gene have, for example, been described for alternative splicing to a proximal or distal splice site in VEGF-A exon 8 producing the pro-angiogenic (VEGF-Axxx, left) and anti-angiogenic (VEGF-Axxxb, right) mRNA isoform families [42]. Furthermore, short peptides derived from CAPERα isoforms a and b were shown to have different and each individually even cell line, as mentioned previously [9].

In the context of integrin α2 expression the differential transactivatory activity of CAPERα isoforms may, for example, apply to the variants of the MII-2 domain (red in Fig. 1c), since the amino acids missing in MM isoforms b, d and g as well as HS isoforms b, d and e (containing either ΔE392-I397 or ΔG366-I397) fall within the domain known to interact with oestrogen receptor α and β [14], which is also implicated in the transcriptional regulation of integrin α2 expression [15, 16], and since the short peptides derived from this region were shown to have differential activity [9].

The sqRT-PCR data obtained here indicates that the full length isoform (MM and HS isoform a) and the isoforms lacking 6 amino acids in the MII-2 region (ΔE392-I397; mainly MM and HS isoform b) predominate in all cell lines tested. Furthermore, lower level expression of variants containing shortened or truncated n-terminal domains (M1-K33sub25 and ΔM1-C157) for both MM cell lines MC3T3 and eEnd2 were detected at approximately equal levels.

Whilst some small differences in the isoform distribution were detected especially between Saos-2 cells and HUVECs, these do not sufficiently explain the observed difference in integrin α2 expression in response to E7820 stimulation, especially since no marked differences could be detected between the expression in MC3T3 cells and HUVECs, which show the greatest difference in response to E7820. Therefore, this data on the relative expression of the CAPERα isoforms together with the data on its degradation in the different cell lines in response to E7820 incubation makes it seem likely that the reason for the observed differing effects of E7820 on integrin α2 is entirely downstream of CAPERα. This may be explained by cell type specific differences in the transactivatory activity of CAPERα on AP-1 via c-Jun, which has previously been hinted at [9]. Simultaneously, the promoter structure of integrin α2 and its previously described cell-type specific functionality would also bear out differential activity of CAPERα via AP-1 and ER [15, 16].

Overall the data presented here in concert with previously published results shows that the regulation of integrin α2 expression by E7820 should be thought of as a multi-step process. The first step is the targeting of CAPERα for proteasomal degradation [12] via a SPLINTS-typical mechanism [10], which appears to be conserved between different both murine and human cell lines. The second step in the regulatory process concerns the co-activatory activity of CAPERα via AP-1 and ER; both the interaction of CAPERα with the c-Jun component of AP-1 and the composition of the Jun-Fos dimer in AP-1 have previously been shown to be cell line specific [9, 20]. The third and final step is the interaction of these factors with the integrin α2 promoter region [9, 14–16]. Analysis of the functionality of the integrin α2 promoter and its interaction with silencer and enhancer elements in osteoblastic cells, however, is at present still lacking.

From a translational standpoint it is important to recognise that the alteration of the expression of cellular adhesion molecules is an important process in tumourigenesis and tumour progression [43]. Various integrins, including integrin α2β1, have been shown to play vital roles in angiogenesis [44] and integrin α2β1 in particular has been shown to facilitate invasion of hepatocarcinoma cells [45]. Furthermore, its increased cell surface expression has been shown to increase proliferation and invasion of various cancer cell lines [46] and it was found to be over expressed in primary colon tumours and their liver metastases [47]. Furthermore, CAPERα has been shown to play a role in the isoform shift from VEGF_189_ to VEGF_164_ in the Ewing sarcoma cell line TC-71 [48]. Here decreased CAPERα levels were associated with a greater shift towards VEGF_164_ and over expression of CAPERα by transfection led to reduced tumour vessel density and growth [48], which indicates that E7820 may result in increased tumour vascularisation in this entity. Since E7820, as we have demonstrated, can lead to increased integrin α2 expression in certain cell lines whilst the proteasomal degradation of CAPERα, which the former induces, is conserved, it may not be suitable for the treatment of malignancies of such an origin. Whilst the data presented herein does not definitively delineate which tumour entities may respond to E7820 with an increase in integrin α2 expression, it seems prudent to consider the possibility of such aberant activity when considering the therapeutic use of E7820 in related malignancies.

## Conclusion

In summary, we were able to illustrate the first published instance of up-regulation of integrin α2 expression by the aromatic sulphonamide E7820. We showed a significant up-regulation of integrin α2 in the MC3T3 osteoblast precursor cell line on both the mRNA and the protein level whilst confirming the expected decrease in CAPERα levels in both MC3T3 cells and HUVECs. Therefore, we can conclude that CAPERα does not positively regulate integrin α2 expression in MC3T3 cells. A conclusive delineation of the genesis of the described up-regulation in integrin α2 expression in MC3T3 cells was, however, not possible.

In the translational context of the increasing number of trials performed using E7820 in cancer therapies this data indicates that it seems to be important to differentiate between those malignancies that will likely respond to E7820 with a reduction in integrin α2 expression from those that may respond with its increase. Although the clinical impact of this effect remains unclear yet, the possibility of entity and cell type specific activity of E7820 should be considered when investigating E7820 both in murine models and clinical trials.

## Supplementary Information

**Additional file 1: Table S1.** Primer sequences for quantitative reverse transcription PCR (qRTPCR) of glycerinaldehyd-3-phosphat-dehydrogenase (GAPDH), hypoxanthinguanin-phosphoribosyltransferase (HPRT), integrin (Itg) α1, α2, α10 and α11, co-activator of AP-1 and ER (CAPERα) and DDB-1 and Cul-4 associated factor 15 (DCAF15). **Table S2.** Forward and reverse primer sequences (top and bottom) and PCR product lengths in base pairs (bp) for semi-quantitative reverse transcription PCR (sqRT-PCR) of CAPERa MII-2 and n-terminal regions. ΔE392-I397 is a 6 amino acid (EFSFVI) deletion and ΔG366-I397 is a 32 amino acid deletion in the MII-2 region; ΔM1-C157 is a deletion of the 157 n-terminal amino acids; M1-K33sub25 is the substitution of the 33 n-terminal amino acids for a different 25 amino acid fragment; DS110-S121 is a 22 amino acid deletion and ΔS121 is a 1 amino acid deletion in the n-terminal domain. These variants of the MII-2 and n-terminal regions combine to form the different isoforms of CAPERα in the manner summarised in Fig. 5 and in supplementary Table S3. **Fiure S1.** Full western blot of integrin a2 (green) and GAPDH (red) correspondingto the bands shown in Fig. 2. **Figure S2.** Full western blot of CAPERa (green) and GAPDH (red) corresponding to the the bands shown in Fig. 3. **Table S3.** CAPERα mRNA and protein isoforms as deposited to the NCBI Gene database for *Mus musculus* (MM) and *Homo sapiens* (HS). The underlined exon indicates the location of the start codon; 1′ is an alternate first exon; 9′ and 16′ are truncated exons resulting from alternate splicing.

## Data Availability

All data generated or analysed during this study are included in this published article [and its supplementary information files].

## Abbreviations

SPLINTS: Small-molecule protein ligand interface stabilisers
E7820: N-(3-Cyano-4-methyl-1H-indol-7-yl)-3-cyanobenzene-sulfonamide)
HUVEC: Human umbilical vein endothelial cells
DDB1: DNA damage binding protein 1
AP-1: Activating protein 1
HEPES: 4-(2-Hydroxyethyl)-piperazin-1-ethansulfonsäure, N-(2-Hydroxyethyl)piperazine-N′-(2-ethanesulfonic acid)
MEM: Minimum essential medium
DMEM: Dulbecco’s modified eagle medium
FBS: Fetal bovine serum
PMA: Phorbol 12-myristate 13-acetate
MTT: 3-(4,5-dimethylthiazol-2-yl)-2,5-diphenyltetrazolium bromide
SDS: Sodium dodecyl sulfate
qRT-PCR: Quantitative real-time polymerase chain reaction
HRPT: Hypoxanthine phosphoribosyl transferase
TBS: Tris buffered saline
BSA: Bovine serum albumn
hs: *Homo sapiens*
mm: *Mus musculus*
VEGF: Vascular endothelial growth factor
ER: Oestrogen receptor
ASC-2: Activating signal co-integrator 2
RRM: RNA-recognition motif

## References

[1] Fink EC, Ebert BL (2015). The novel mechanism of lenalidomide activity. Blood..

[2] Valeur E, Narjes F, Ottmann C, Plowright AT (2019). Emerging modes-of-action in drug discovery. Med Chem Comm.

[3] Fischer ES, Park E, Eck MJ, Thomä NH (2016). SPLINTS: small-molecule protein ligand interface stabilizers. Curr Opin Struct Biol.

[4] Funahashi Y, Sugi NH, Semba T, Yamamoto Y, Hamaoka S, Tsukahara-Tamai N, Ozawa Y, Tsuruoka A, Nara K, Takahashi K, Okabe T, Kamata J, Owa T, Ueda N, Haneda T, Yonaga M, Yoshimatsu K, Wakabayashi T (2002). Sulfonamide derivative, E7820, is a unique angiogenesis inhibitor suppressing an expression of integrin alpha2 subunit on endothelium. Cancer Res.

[5] Semba T, Funahashi Y, Ono N, Yamamoto Y, Sugi NH, Asada M, Yoshimatsu K, Wakabayashi T (2004). An angiogenesis inhibitor E7820 shows broad-Spectrum tumor growth inhibition in a xenograft model: possible value of integrin α2 on platelets as a biological marker. Clin Cancer Res.

[6] Sawyer MB, Iqbal S, Lenz H, Lima CSR, Rossignol DP, Krivelevich I (2010). Phase II study of E7820 in combination with cetuximab in subjects (pts) with metastatic and refractory colorectal cancer (CRC). J Clin Oncol.

[7] Mita M, Kelly KR, Mita A, Ricart AD, Romero O, Tolcher A, Hook L, Okereke C, Krivelevich I, Rossignol DP, Giles FJ, Rowinsky EK, Takimoto C (2011). Phase I study of E7820, an Oral inhibitor of integrin α-2 expression with antiangiogenic properties, in patients with advanced malignancies. Clin Cancer Res.

[8] Kerklaan BM, Slater S, Flynn M, Greystoke A, Witteveen PO, Megui-Roelvink M (2016). A phase I, dose escalation, pharmacodynamic, pharmacokinetic, and food-effect study of α2 integrin inhibitor E7820 in patients with advanced solid tumors. Investig New Drugs.

[9] Chilewski SD, Bhosale D, Dees S, Hutchinson I, Trimble R, Pontiggia L, Mercier I, Jasmin JF (2020). Development of CAPER peptides for the treatment of triple negative breast cancer. Cell Cycle Taylor & Francis.

[10] Faust TB, Yoon H, Nowak RP, Donovan KA, Li Z, Cai Q (2019). Structural complementarity facilitates E7820-mediated degradation of RBM39 by DCAF15. Nat Chem Biol.

[11] Bier D, Thiel P, Briels J, Ottmann C (2015). Stabilization of protein–protein interactions in chemical biology and drug discovery. Prog Biophys Mol Biol Elsevier Ltd.

[12] Uehara T, Minoshima Y, Sagane K, Sugi NH, Mitsuhashi KO, Yamamoto N, Kamiyama H, Takahashi K, Kotake Y, Uesugi M, Yokoi A, Inoue A, Yoshida T, Mabuchi M, Tanaka A, Owa T (2017). Selective degradation of splicing factor CAPERα by anticancer sulfonamides. Nat Chem Biol.

[13] Petroski MD, Deshaies RJ (2005). Function and regulation of cullin–RING ubiquitin ligases. Nat Rev Mol Cell Biol.

[14] Jung D-J, Na S-Y, Na DS, Lee JW (2002). Molecular cloning and characterization of CAPER, a novel coactivator of activating Protein-1 and estrogen receptors. J Biol Chem.

[15] Zutter MM, Santoro SA, Painter AS, Tsung YL, Gafford A (1994). The human alpha 2 integrin gene promoter. Identification of positive and negative regulatory elements important for cell-type and developmentally restricted gene expression. J Biol Chem.

[16] Zutter MM, Painter AA, Staatz WD, Tsung YL (1995). Regulation of alpha 2 integrin gene expression in cells with megakaryocytic features: a common theme of three necessary elements. Blood..

[17] Han T, Goralski M, Gaskill N, Capota E, Kim J, Ting TC (2017). Anticancer sulfonamides target splicing by inducing RBM39 degradation via recruitment to DCAF15. Science (80- ).

[18] Sang Y, Yan F, Ren X (2015). The role and mechanism of CRL4 E3 ubiquitin ligase in cancer and its potential therapy implications. Oncotarget..

[19] O’Leary NA, Wright MW, Brister JR, Ciufo S, Haddad D, McVeigh R (2016). Reference sequence (RefSeq) database at NCBI: current status, taxonomic expansion, and functional annotation. Nucleic Acids Res.

[20] Angel P, Karin M (1991). The role of Jun, Fos and the AP-1 complex in cell-proliferation and transformation. BBA - Rev Cancer.

[21] Cheli Y, Kanaji S, Jacquelin B, Chang M, Nugent DJ, Kunicki TJ (2007). Transcriptional and epigenetic regulation of the integrin collagen receptor locus ITGA1-PELO-ITGA2. Biochim Biophys Acta - Gene Struct Expr.

[22] Wu X, Cai J, Zuo Z, Li J (2019). Collagen facilitates the colorectal cancer stemness and metastasis through an integrin/PI3K/AKT/snail signaling pathway. Biomed Pharmacother.

[23] Ito K, Semba T, Uenaka T, Wakabayashi T, Asada M, Funahashi Y (2014). Enhanced anti-angiogenic effect of E7820 in combination with erlotinib in epidermal growth factor receptor-tyrosine kinase inhibitor-resistant non-small-cell lung cancer xenograft models. Cancer Sci.

[24] Chung CH, Chang CH, Hsu CC, Lin KT, Peng HC, Huang TF (2017). Aggretin Venom Polypeptide as a Novel Anti-angiogenesis Agent by Targeting Integrin alpha2beta1. Sci Rep.

[25] Stange R, Kronenberg D, Timmen M, Everding J, Hidding H, Eckes B, Hansen U, Holtkamp M, Karst U, Pap T, Raschke MJ (2013). Age-related bone deterioration is diminished by disrupted collagen sensing in integrin α2β1 deficient mice. Bone..

[26] Wang D, Christensen K, Chawla K, Xiao G, Krebsbach PH, Franceschi RT (1999). Isolation and characterization of MC3T3-E1 Preosteoblast subclones with distinct in vitro and in vivo differentiation/mineralization potential. J Bone Miner Res.

[27] Rodan SB, Imai Y, Thiede MA, Wesolowski G, Thompson D, Bar-Shavit Z, Shull S, Mann K, Rodan GA (1987). Characterization of a human osteosarcoma cell line (Saos-2) with osteoblastic properties. Cancer Res.

[28] Williams RL, Risau W, Zerwes H-G, Drexler H, Aguzzi A, Wagner EF (1989). Endothelioma cells expressing the polyoma middle T oncogene induce hemangiomas by host cell recruitment. Cell..

[29] Filipovic N, Ghimire K, Saveljic I, Milosevic Z, Ruegg C (2016). Computational modeling of shear forces and experimental validation of endothelial cell responses in an orbital well shaker system. Comput methods Biomech biomed Engin. Taylor Francis.

[30] Goel G, Makkar HPS, Francis G, Becker K (2007). Phorbol esters: structure, biological activity, and toxicity in animals. Int J Toxicol.

[31] Mosmann T (1983). Rapid colorimetric assay for cellular growth and survival: application to proliferation and cytotoxicity assays. J Immunol Methods.

[32] Livak KJ, Schmittgen TD (2001). Analysis of relative gene expression data using real-time quantitative PCR and the 2-ΔΔCT method. Methods..

[33] Kronke J, Udeshi ND, Narla A, Grauman P, Hurst SN, McConkey M (2014). Lenalidomide Causes Selective Degradation of IKZF1 and IKZF3 in Multiple Myeloma Cells. Science (80- ).

[34] Gullberg DE, Lundgren-Åkerlund E (2002). Collagen-binding I domain integrins — what do they do?. Prog Histochem Cytochem.

[35] Kronenberg D, Michel PA, Hochstrat E, Wei M, Brinckmann J, Müller M (2020). Increased collagen turnover impairs tendon microstructure and stability in integrin α2β1-deficient mice. Int J Mol Sci.

[36] Obata H, Hayashi K, Nishida W, Momiyama T, Uchida A, Ochi T (1997). Smooth muscle cell phenotype-dependent transcriptional regulation of the alpha1 integrin gene. J Biol Chem.

[37] Vigneault F, Zaniolo K, Gaudreault M, Gingras M-E, Guérin SL (2007). Control of integrin genes expression in the eye. Prog Retin Eye Res.

[38] Lu N, Heuchel R, Barczyk M, Zhang W-M, Gullberg D (2006). Tandem Sp1/Sp3 sites together with an Ets-1 site cooperate to mediate alpha11 integrin chain expression in mesenchymal cells. Matrix Biol.

[39] Lu N, Carracedo S, Ranta J, Heuchel R, Soininen R, Gullberg D (2010). The human alpha11 integrin promoter drives fibroblast-restricted expression in vivo and is regulated by TGF-beta1 in a Smad- and Sp1-dependent manner. Matrix Biol.

[40] Xu J, Zutter MM, Santoro SA, Clark RA (1998). A three-dimensional collagen lattice activates NF-kappaB in human fibroblasts: role in integrin alpha2 gene expression and tissue remodeling. J Cell Biol.

[41] Engel BE, Welsh E, Emmons MF, Santiago-Cardona PG, Cress WD (2013). Expression of integrin alpha 10 is transcriptionally activated by pRb in mouse osteoblasts and is downregulated in multiple solid tumors. Cell Death Dis.

[42] Harper SJ, Bates DO (2008). VEGF-A splicing: the key to anti-angiogenic therapeutics?. Nat Rev Cancer.

[43] Läubli H, Borsig L (2019). Altered cell adhesion and glycosylation promote Cancer immune suppression and metastasis. Front Immunol.

[44] Desgrosellier JS, Cheresh DA (2010). Integrins in cancer: biological implications and therapeutic opportunities. Nat Rev Cancer.

[45] Yang C, Zeisberg M, Lively JC, Nyberg P, Afdhal N, Kalluri R (2003). Integrin alpha1beta1 and alpha2beta1 are the key regulators of hepatocarcinoma cell invasion across the fibrotic matrix microenvironment. Cancer Res.

[46] Ren D, Zhao J, Sun Y, Li D, Meng Z, Wang B (2019). Overexpressed ITGA2 promotes malignant tumor aggression by up-regulating PD-L1 expression through the activation of the STAT3 signaling pathway. J Exp Clin Cancer Res.

[47] Yang Q, Bavi P, Wang JY, Roehrl MH (2017). Immuno-proteomic discovery of tumor tissue autoantigens identifies olfactomedin 4, CD11b, and integrin alpha-2 as markers of colorectal cancer with liver metastases. J Proteomics.

[48] Huang G, Zhou Z, Wang H, Kleinerman ES (2012). CAPER-α alternative splicing regulates the expression of vascular endothelial growth factor165 in Ewing sarcoma cells. Cancer..

