# Supplementary material for: The small-molecule protein ligand interface stabiliser E7820 induces differential cell line specific responses of integrin α2 expression
Source: BMC Cancer. 2021 May 18;21:571. doi: 10.1186/s12885-021-08301-w (PMC8132423; doi:10.1186/s12885-021-08301-w)
Supplement: Supplementary file 1 — Additional file 1: Table S1. Primer sequences for quantitative reverse transcription PCR (qRTPCR) of glycerinaldehyd-3-phosphat-dehydrogenase (GAPDH), hypoxanthinguanin-phosphoribosyltransferase (HPRT), integrin (Itg) α1, α2, α10 and α11, co-activator of AP-1 and ER (CAPERα) and DDB-1 and Cul-4 associated factor 15 (DCAF15). Table S2. Forward and reverse primer sequences (top and bottom) and PCR product lengths in base pairs (bp) for semi-quantitative reverse transcription PCR (sqRT-PCR) of CAPERa MII-2 and n-terminal regions. ΔE392-I397 is a 6 amino acid (EFSFVI) deletion and ΔG366-I397 is a 32 amino acid deletion in the MII-2 region; ΔM1-C157 is a deletion of the 157 n-terminal amino acids; M1-K33sub25 is the substitution of the 33 n-terminal amino acids for a different 25 amino acid fragment; DS110-S121 is a 22 amino acid deletion and ΔS121 is a 1 amino acid deletion in the n-terminal domain. These variants of the MII-2 and n-terminal regions combine to form the different isoforms of CAPERα in the manner summarised in Fig. 5 and in supplementary Table S3. Fiure S1. Full western blot of integrin a2 (green) and GAPDH (red) correspondingto the bands shown in Fig. 2. Figure S2. Full western blot of CAPERa (green) and GAPDH (red) corresponding to the the bands shown in Fig. 3. Table S3. CAPERα mRNA and protein isoforms as deposited to the NCBI Gene database for Mus musculus (MM) and Homo sapiens (HS). The underlined exon indicates the location of the start codon; 1′ is an alternate first exon; 9′ and 16′ are truncated exons resulting from alternate splicing. [file 12885_2021_8301_MOESM1_ESM.pdf]

The small-molecule protein ligand interface stabiliser E7820 induces differential cell line specific responses of integrin α2 expression

Michael David Hülskamp, Daniel Kronenberg, Richard Stange

-supplemental data -

Mus musculus

Target	Forward	Reverse
GAPDH	tgtgcagtgccagcctcgtc	ctcggccttgactgtgccgt
HPRT	tgatagatccattcctatga	aagacattctttccagttaa
Itga2	ctatggccacgtcagcaatg	gctttggggcaagtgattca
Itga1	gatggggacgtcaacattct	tgtggttaagacgctaccaaag
Itga10	gaatcaggccgcatcctac	aagtatcggagggcctgtg
Itga11	gcagacgtcctctttaccaga	gagctgtttgccttgacctc
CAPERa	gatattcgggttgggcggta	ttctctactcgcctgtgcg
DCAF15	agtcaagatcagtgggcagc	ttcttgagggacacgcagac

Homo Sapiens

Target	Forward	Target Forward Reverse
GAPDH	gtctcctctgacttcaacagcg	accaccctgttgctgtagccaa
HPRT	gaccagtcaacaggggacat	gtgtcaattatatcttccacaatcaag
Itga2	gcaactggttactggttggtt	gcaactggttactggttggtt
CAPERa	tgcagagggtacaggtttgc	ccacagcaccaaatgccaaa

Table S1: Primer sequences for quantitative reverse transcription PCR (qRTPCR)of glycerinaldehyd-3-phosphat-dehydrogenase (GAPDH), hypoxanthinguanin-phosphoribosyltransferase (HPRT), integrin (Itg) α1, α2, α10 and α11,co-activator of AP-1 and ER (CAPERα) and DDB-1 and Cul-4 associated factor 15 (DCAF15).

Mus musculus

tgggaacaactggacgtctt	full	ΔE392-I397	
gcaagaggttgaacagacgc	214 bp	196 bp	
gagtagagaaaatggcagacgatat	full	ΔM1-C157	M1-K33sub25
gaaaatgtaccattacatgctggttt	256 bp	456/329 bp	384 bp
cttacaagaaggatgagaacaagttg	ΔS121		
ttcgggaacgtcttcttaatttg	336 bp		

Homo Sapiens

aagcactgtctcctgtcct	full	ΔE392-I397	ΔG366-I397
tggaaaatgcatgaaagtatccctc	398 bp	380 bp	302 bp
cggccatgaagaacgtagca	full	ΔM1-C157	ΔS110-S121
cttggtcgaattcttgccgc	420/423 bp	495 bp	357 bp
gcctcatagcatcaaattaagacg	ΔS121		
gcctgtgatgcctgtactatg	345 bp		

Table S2: Forward and reverse primer sequences (top and bottom) and PCR product lengths in base pairs (bp) for semi-quantitative reverse transcription PCR (sqRT-PCR) of CAPERa MII-2 and n-terminal regions. ΔE392-I397 is a 6 amino acid (EFSFVI) deletion and ΔG366-I397 is a 32 amino acid deletion in the MII-2 region; ΔM1-C157 is a deletion of the 157 n-terminal amino acids; M1-K33sub25 is the substitution of the 33 n-terminal amino acids for a different 25 amino acid fragment; DS110-S121 is a 22 amino acid deletion and ΔS121 is a 1 amino acid deletion in the n-terminal domain. These variants of the MII-2 and n-terminal regions combine to form the different isoforms of CAPERα in the manner summarised in figure 5 and in supplementary table S3.

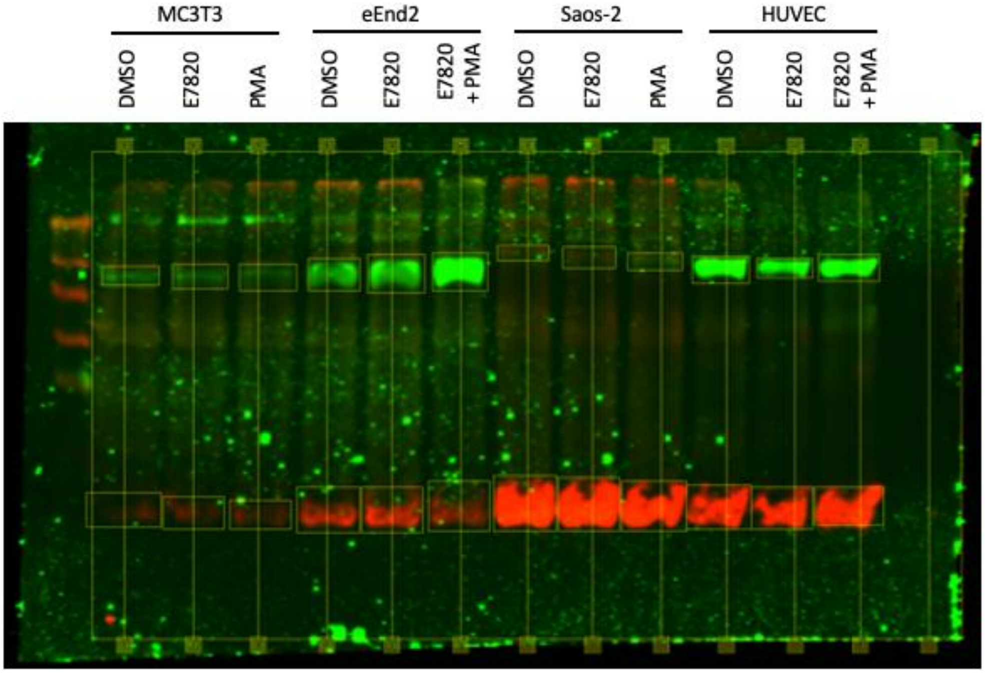

Figure S1: Full western blot of integrin a2 (green) and GAPDH (red) correspondingto the bands shown in figure 2.

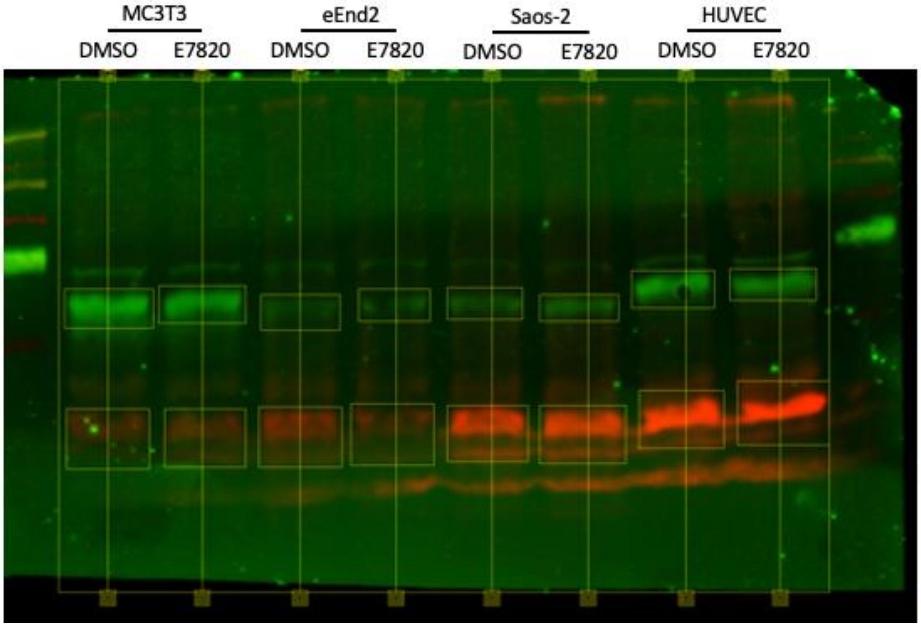

Figure S2: Full western blot of CAPERa (green) and GAPDH (red) corresponding to the the bands shown in figure 3.

	mRNA			protein		
Species	Accession	Isoform	Exon structure	Accession	Isoform	Characteristics
HS	NM_184234.3	1	1-2-4-7-8-9-10-11-12-13-14-15-16-17-18-19-20	NP_909122.1	a	full length
MM	NM_133242.3	1		NP_573505.2	a	
HS	NM_004902.4	2	1-2-4-7-8-9-10-11-12-13-14-15-16’-17-18-19-20	NP_004893.1	b	ΔE392-I397
MM	NM_001291114.1	2		NP_001278043.1	b	
MM	M_001291115.1	3	1’-2-4-7-8-9-10-11-12-13-14-15-16’-17-18-19-20	NP_001278044.1		
HS	NM_001242599.2	3	1-2-4-7-9’-10-11-12-13-14-15-16-17-18-19-20	NP_001229528.1	c	ΔS100-S121
HS	NM_001242600.2	4	1-2-4-7-9’-10-11-12-13-14-15-16’-17-18-19-20	NP_001229529.1	d	ΔS100-S121, ΔE392-I397
HS	NM_001323422.2	8	1-2-4-7-8-9-10-11-12-13-14-16’-17-18-19-20	NP_001310351.1	e	ΔG366-I397
HS	NM_001323423.2	9	1-2-3-4-6-7-8-9-10-11-12-13-14-15-16-17-18-19-20	NP_001310352.1	f	ΔM1-C157
MM	NM_001362766.1	7	1-2-3-4-5-6-7-8-9-10-11-12-13-14-15-16-17-18-19-20	NP_001349695.1		
MM	NM_001362767.1	8	1-2-3-4-7-8-9-10-11-12-13-14-15-16-17-18-19-20	NP_001349696.1	f	
MM	NM_001362768.1	9	1-2-3-4-7-8-9’-10-11-12-13-14-15-16-17-18-19-20	NP_001349697.1		
HS	NM_001323424.2	10	1-2-4-7-8-9’-10-11-12-13-14-15-16-17-18-19-20	NP_001310353.1	g	ΔM1-C157
MM	NM_001362763.1	4		NP_001349692.1	c	
MM	NM_001362764.1	5	1-2-4-7-8-9’-10-11-12-13-14-15-16-17-18-19-20	NP_001349693.1	d	ΔS121, ΔE392-I397
MM	NM_001362765.1	6	1-2-4-7-8-9’-10-11-12-13-14-15-16’-17-18-19-20	NP_001349694.1	e	M1-K33sub25AA
MM	NM_001362765.1	10	1-2-3-4-7-8-9-10-11-12-13-14-15-16’-17-18-19-20	NP_001349698.1	g	ΔM1-C157, ΔE392-I397

Table S3: CAPERα mRNA and protein isoforms as deposited to the NCBI Gene database for Mus musculus (MM) and Homo sapiens (HS). The underlined exon indicates the location of the start codon; 1’ is an alternate first exon; 9’ and 16’ are truncated exons resulting from alternate splicing
